# Characteristics and Clinical Outcomes of Patients with Chronic Lymphocytic Leukemia/Small Lymphocytic Lymphoma Receiving Ibrutinib for ≥5 Years in the RESONATE-2 Study

**DOI:** 10.3390/cancers15020507

**Published:** 2023-01-13

**Authors:** Jennifer A. Woyach, Paul M. Barr, Thomas J. Kipps, Jacqueline C. Barrientos, Inhye E. Ahn, Paolo Ghia, Vincent Girardi, Emily Hsu, Mandy Jermain, Jan A. Burger

**Affiliations:** 1 Ohio State University Comprehensive Cancer Center, Columbus, OH 43210, USA; 2 Wilmot Cancer Institute, University of Rochester, Rochester, NY 14642, USA; 3 University of California San Diego Moores Cancer Center, San Diego, CA 92037, USA; 4 Mount Sinai Comprehensive Cancer Center, Miami Beach, FL 33140, USA; 5 Laboratory of Lymphoid Malignancies, National Heart, Lung, and Blood Institute, National Institutes of Health, Bethesda, MD 20814, USA; 6 Università Vita-Salute San Raffaele and IRCCS Ospedale San Raffaele, 20132 Milan, Italy; 7 Pharmacyclics LLC, an AbbVie Company, South San Francisco, CA 94080, USA; 8 University of Texas MD Anderson Cancer Center, Houston, TX 77030, USA

**Keywords:** ibrutinib, chronic lymphocytic leukemia, long-term outcomes

## Abstract

**Simple Summary:**

Ibrutinib is an established standard of care in the first-line treatment of chronic lymphocytic leukemia. Since ibrutinib is given continuously, data on long-term treatment, outcomes, and safety are essential to inform clinical decision making. Here, we describe characteristics and outcomes of patients who received long-term treatment with ibrutinib for ≥5 years in the phase 3 RESONATE-2 study. More than half (58%) of the patients randomly assigned to receive ibrutinib in the RESONATE-2 study continued to benefit from ibrutinib treatment for ≥5 years, regardless of baseline characteristics. Among patients who were on ibrutinib treatment for ≥5 years, complete response rates improved over time through the 5 years. The safety profile of ibrutinib treatment for ≥5 years was consistent with previous reports and no new safety signals were identified. For patients who experienced adverse events, dose modification was effective in resolving adverse events, thereby facilitating continued treatment.

**Abstract:**

Primary results from the phase 3 RESONATE-2 study demonstrated superior efficacy and tolerability with ibrutinib versus chlorambucil in patients with chronic lymphocytic leukemia (CLL)/small lymphocytic lymphoma (SLL). Here, we describe characteristics and outcomes of patients who received ibrutinib treatment for ≥5 years in RESONATE-2. Patients aged ≥65 years with previously untreated CLL/SLL, without del(17p), were randomly assigned 1:1 to once-daily ibrutinib 420 mg until disease progression/unacceptable toxicity (*n* = 136) or chlorambucil 0.5–0.8 mg/kg for ≤12 cycles (*n* = 133). Baseline characteristics in ibrutinib-randomized patients (*n* = 136) were generally similar between patients on ibrutinib treatment for ≥5 years (*n* = 79) versus those on treatment for <5 years (*n* = 57). In patients on ibrutinib treatment for ≥5 years, complete response rates improved over time, reaching 42% by 5 years. Estimated 7-year progression-free survival and overall survival rates were 82% and 94%, respectively. Adverse events (AEs) led to dose reductions in 16/79 patients (20%); these AEs were resolved for 13/16 patients (81%). AEs led to dose holds (≥7 days) in 45/79 patients (57%); these AEs were resolved for 43/45 patients (96%). More than half (58%) of ibrutinib-randomized patients benefitted from ibrutinib treatment for ≥5 years regardless of baseline characteristics. Dose modification resolved AEs for most patients, thereby facilitating continued treatment.

## 1. Introduction

Ibrutinib is a once-daily oral Bruton tyrosine kinase (BTK) inhibitor that is approved as first-line treatment for patients with chronic lymphocytic leukemia (CLL)/small lymphocytic lymphoma (SLL) in the United States, Europe, and other countries. Ibrutinib has the longest follow-up of any targeted therapy across multiple randomized phase 3 studies and is the only therapy to date that has demonstrated a significant overall survival (OS) benefit compared with chemotherapy/chemoimmunotherapy in patients with previously untreated CLL/SLL [1,2,3,4]. Initial approval of ibrutinib in the first-line setting was supported by results from the primary analysis of the phase 3 RESONATE-2 study, which demonstrated that ibrutinib was superior to chlorambucil with respect to both efficacy and tolerability [5]. With up to 8 years of follow-up (median: 82.7 months; range, 0.1–96.6 months) in the RESONATE-2 study, the majority of ibrutinib-randomized patients remained progression-free; median progression-free survival (PFS) was not yet reached at the latest data cut [1].

Previous studies suggest that patients who continue treatment with single-agent ibrutinib experience better survival outcomes than patients who discontinue treatment within the first few years [6,7,8,9]. Additionally, real-world evidence suggests that dose management (dose reduction or temporary dose holds for up to 1–2 weeks) results in improvement in or resolution of adverse events (AEs) [10] without impacting disease outcomes [6,10,11,12,13,14,15,16,17,18]. Therefore, active management of AEs by dose modification might facilitate continued ibrutinib treatment and maximize clinical outcomes [12]. As of May 2022, the US prescribing information for ibrutinib includes updates to recommended dose modifications for AEs [19].

As it is the only BTK inhibitor with long-term follow-up for up to 8 years, we have the opportunity to examine efficacy and safety outcomes in patients with longer-term experience on ibrutinib treatment. Here, we describe characteristics and outcomes of patients who received treatment with ibrutinib for ≥5 years in the RESONATE-2 study.

## 2. Materials and Methods

### 2.1. Study Design and Patients

RESONATE-2 (PCYC-1115 [NCT01722487]/PCYC-1116 [NCT01724346]) is a multicenter, international, randomized, open-label, phase 3 study designed to compare the efficacy and safety of first-line treatment with ibrutinib versus chlorambucil in patients aged ≥65 years with previously untreated CLL/SLL who required therapy per the 2008 International Workshop on CLL (iwCLL) criteria [20]. Patients with del(17p) were excluded. Detailed methods were previously reported [5]. Briefly, eligible patients were randomly assigned in a 1:1 ratio to receive oral ibrutinib 420 mg once daily until occurrence of progressive disease or unacceptable toxicity, or chlorambucil 0.5 mg/kg, escalated to a maximum of 0.8 mg/kg as tolerated, on days 1 and 15 of each 28-day cycle for up to 12 cycles. After confirmation of progressive disease per iwCLL criteria [20,21], patients who were randomly assigned to the chlorambucil arm could cross over to second-line treatment with ibrutinib. Per protocol, ibrutinib was temporarily held for any unmanageable grade ≥ 3 AE that was considered by the investigator to be potentially related to the study’s treatment. Other AEs, including AEs of grade 2 in severity, could be managed with a one-level dose reduction of ibrutinib if the AE was considered to be potentially manageable by dose reduction as judged by the investigator.

This study was performed in accordance with International Conference on Harmonisation Guidelines for Good Clinical Practice and the principles of the Declaration of Helsinki. The study protocol was approved by institutional review boards of each participating institution, and all patients provided written informed consent before participation in the study. This study was registered with ClinicalTrials.gov, numbers NCT01722487 and NCT01724346.

### 2.2. Analysis

The current exploratory analysis evaluated baseline demographics and clinical characteristics, overall response rates (per iwCLL criteria [20,21]), PFS, OS, prevalence of AEs over time, and AEs leading to dose modifications (per protocol) for ibrutinib-randomized patients who were on ibrutinib treatment for ≥5 years. Since the study protocol provided flexibility for dose reductions based on an investigator’s judgment, additional analyses were performed to retrospectively determine the incidence of dose reductions due to AEs for which dose reductions are recommended in the recently updated US prescribing information (grade 2 cardiac failure, grade 3 cardiac arrhythmia, grade 3 or 4 nonhematologic AEs [excluding cardiac failure and cardiac arrhythmia], grade 3 or 4 neutropenia with infection or fever, and grade 4 hematologic AEs) [19].

Baseline characteristics were also evaluated as potential predictors for remaining on treatment for ≥5 years using a multivariate logistic regression model including the following baseline characteristics: age group, sex, Eastern Cooperative Oncology Group performance status, Cumulative Illness Rating Scale score, creatinine clearance, *TP53* mutation status, IGHV mutation status, del(11q) status, disease histology, bulky disease, β-2 microglobulin, Rai stage, any history of cytopenia, lactate dehydrogenase level, and geographic region. Additionally, PFS and OS were analyzed in subgroups of patients with and without dose reductions in the overall population of all ibrutinib-treated patients; these exploratory post hoc analyses were not powered for significance, and comparative statistics are provided for descriptive purposes only. PFS and OS were estimated using the Kaplan-Meier method.

## 3. Results

In RESONATE-2, 269 patients were randomly assigned to receive ibrutinib (*n* = 136) or chlorambucil (*n* = 133). Of the 136 patients in the ibrutinib arm, 79 (58%) received ibrutinib treatment for ≥5 years. Median follow-up duration for patients who were on ibrutinib treatment for ≥5 years (*n* = 79) was 89.2 months (range: 61.3–96.6). Of these 79 patients, 22 subsequently discontinued ibrutinib in years 5–6 (*n* = 9), 6–7 (*n* = 10), or 7–8 (*n* = 3); reasons for discontinuation in these 22 patients were progressive disease (*n* = 10), death (*n* = 4), AEs (*n* = 3), physician decision (*n* = 3), and patient withdrawal (*n* = 2).

### 3.1. Baseline Characteristics

Within ibrutinib-randomized patients in the intention-to-treat population (*n* = 136), baseline characteristics in the subset of patients who were on ibrutinib treatment for ≥5 years (*n* = 79) were generally similar to those in the subset of patients who were on ibrutinib treatment for <5 years (*n* = 57) (Table 1). Compared with the subset of patients who were on ibrutinib treatment for <5 years, the subset of patients who were on ibrutinib treatment for ≥5 years were more likely to be in the youngest age group (65–69 years; 37% vs. 19% of patients) and had a longer interval between initial diagnosis and initiation of study treatment (median 35 vs. 26 months).

### 3.2. Predictors of Ibrutinib Treatment for ≥5 Years

**Figure 1:**
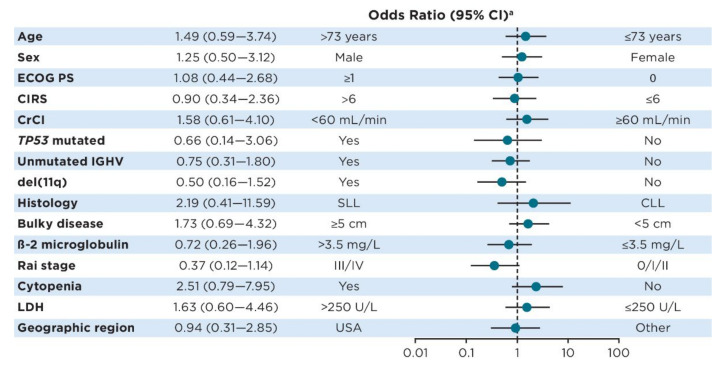
Forest plot of odds for continued ibrutinib treatment at 5 years according to baseline characteristics. ^a^ Odds ratios < 1 favor achievement of ≥5 years of ibrutinib treatment for the subgroup to the left of the plot, while odds ratios > 1 favor achievement of ≥5 years of ibrutinib treatment for the subgroup to the right of the plot. Abbreviations: CIRS, Cumulative Illness Rating Scale; CrCl, creatinine clearance; ECOG PS, Eastern Cooperative Oncology Group performance status; IGHV, immunoglobulin heavy chain variable region; LDH, lactate dehydrogenase.

In multivariate analysis, several baseline characteristics showed a trend toward continuation of ibrutinib treatment for ≥5 years (age ≤ 73 years, female sex, creatinine clearance ≥60 mL/min, *TP53* mutated, del(11q), CLL histology, absence of bulky disease [<5 cm], β-2 microglobulin >3.5 mg/L, Rai stage III/IV, absence of cytopenia, and lactate dehydrogenase ≤250 U/L), but none reached statistical significance (Figure 1).

### 3.3. Efficacy in Patients on Ibrutinib Treatment for ≥5 Years

**Figure 2:**
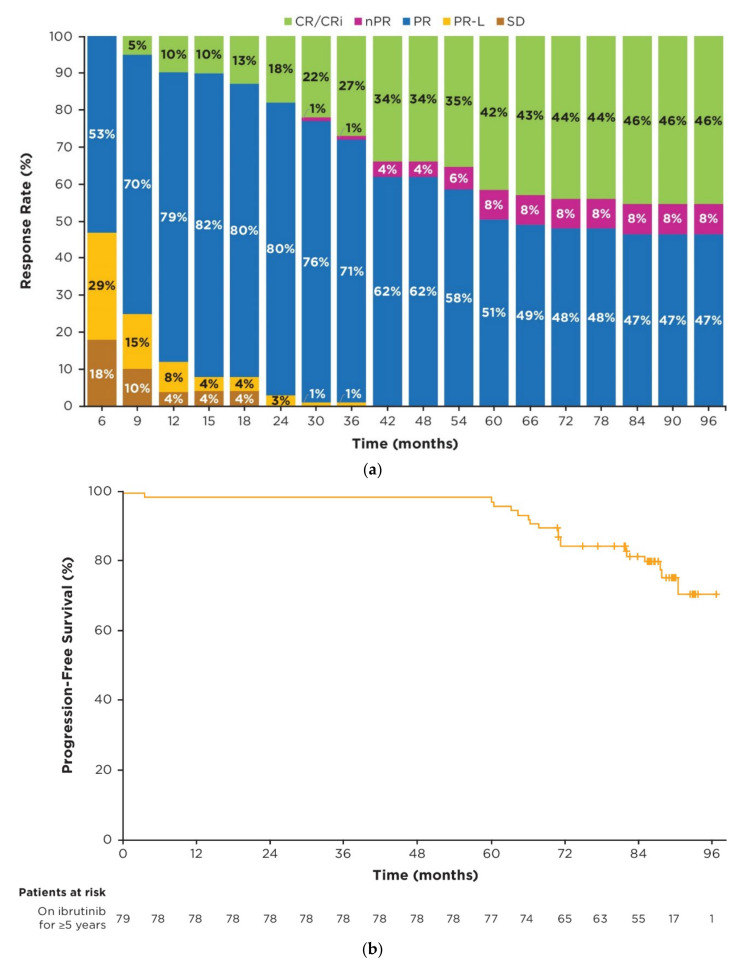

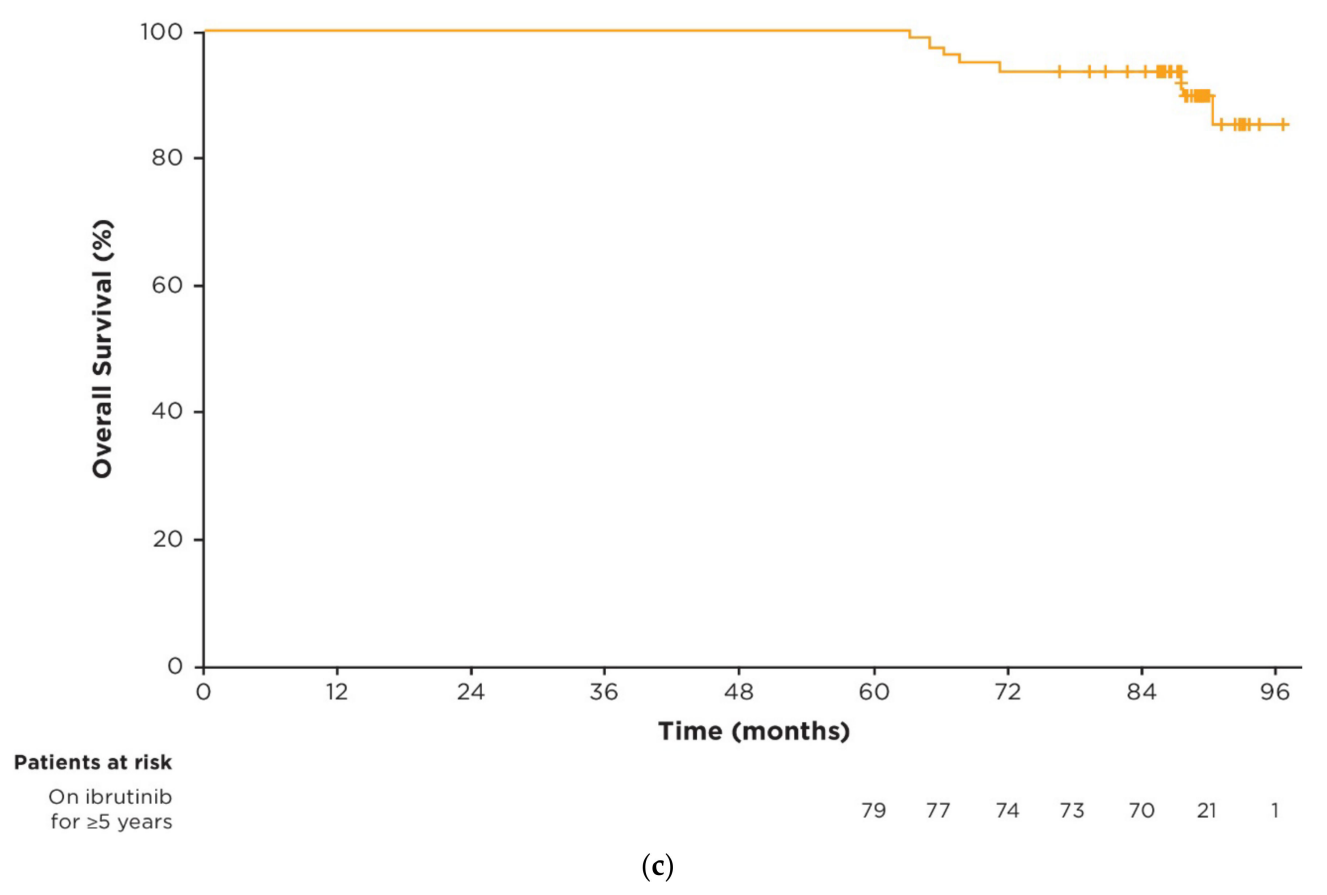
Efficacy in patients on ibrutinib treatment for ≥5 years (*n* = 79). (**a**) Cumulative best response over time; (**b**) Kaplan–Meier curve of investigator-assessed PFS; (**c**) Kaplan–Meier curve of OS. Percentages of patients in each category of response may not add up to the overall proportion with a response due to rounding. Abbreviations: CR, complete response; CRi, complete response with incomplete bone marrow recovery; nPR, nodular partial response; OS, overall survival; PFS, progression-free survival; PR, partial response; PR-L, partial response with lymphocytosis; SD, stable disease.

Responses deepened over time, as indicated by the improvement of complete response (CR) rates from 10% (8/79 patients) at 1 year to 42% (33/79 patients) by 5 years and 46% (36/79 patients) by 7 years (Figure 2a). In patients who were on ibrutinib treatment for ≥5 years, 23/79 (29%) had a documented response of partial response with lymphocytosis (PR-L); of these patients, 9/23 (39%) achieved a best response of partial response (PR), 1/23 (4%) achieved nodular PR (nPR), and 13/23 (57%) achieved CR. In the overall population of ibrutinib-randomized patients, 30/136 (22%) had a documented response of PR-L; of these patients, 13/30 (43%) achieved a best response of PR, 1/30 (3%) achieved nPR, and 15/30 (50%) achieved CR. In patients who were on ibrutinib treatment for ≥5 years, the median time to PR was 4.6 months (95% CI: 3.8–7.4), whereas the median time to CR was 32.3 months (95% CI: 19.7–37.7) for those patients achieving CR. With up to 8 years of follow-up, complete response was achieved in 44 patients in the overall population, 36 of whom received ibrutinib treatment for ≥5 years.

In patients who were on ibrutinib treatment for ≥5 years, median PFS and OS were not yet reached; 7-year PFS and OS rates were 82% (95% CI: 71–89) and 94% (95% CI: 86–97), respectively (Figure 2b,c).

### 3.4. Prevalence of AEs over Time

**Figure 3:**
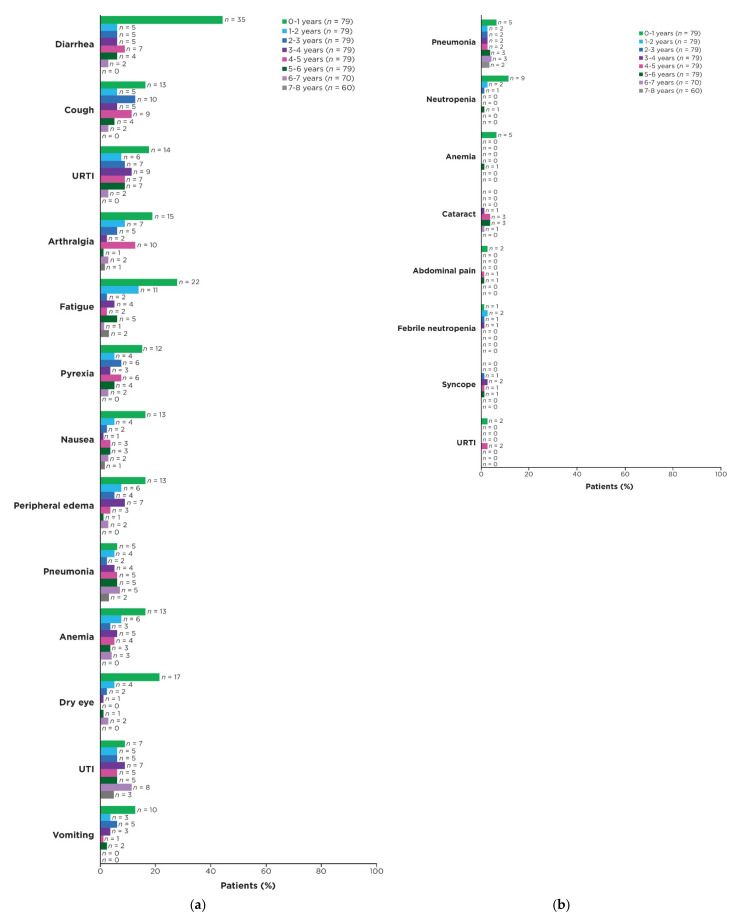
Prevalence of AEs over time in patients on ibrutinib treatment for ≥5 years. (**a**) Most frequent AEs of any grade (occurring in ≥25% of patients overall) by yearly interval; (**b**) Most frequent grade ≥ 3 AEs (occurring in ≥5% of patients overall) by yearly interval. Prevalence was determined by the proportion of patients with a given AE (existing event or new onset of an event) during each yearly interval. Multiple onsets of the same AE term within a specific yearly interval were counted once, and the same AE term continuing across several yearly intervals was counted in each of the intervals. Abbreviations: AE, adverse event; UTI, urinary tract infection; URTI, upper respiratory tract infection.

In patients who were on ibrutinib treatment for ≥5 years, the median duration of ibrutinib treatment was 89.2 months (range: 60.4–96.6). Median relative dose intensity of ibrutinib for these patients was 98% (range: 47–100). The most frequent AEs of any grade across the entire study period were diarrhea (42/79 patients; 53%), cough (34/79; 43%), and upper respiratory tract infection (33/79; 42%). Prevalence of the most frequent AEs of any grade and of grade ≥ 3 were generally highest in years 0–1 and decreased over time thereafter (Figure 3a,b). Prevalence of AEs of clinical interest of any grade over time are shown in Supplementary Figure S1.

### 3.5. Dose Management with Ibrutinib Treatment

AEs led to dose reductions in 16/79 patients (20%) who were on ibrutinib treatment for ≥5 years and in 31/135 patients (23%) in the overall population of all ibrutinib-treated patients (Table 2). Most patients (12/16; 75%) experienced only one AE leading to dose reduction.

Among patients who were on ibrutinib treatment for ≥5 years, the lowest ibrutinib dose for most patients with dose reductions was 280 mg once daily (10/16 patients). At data cutoff, 3/16 patients were receiving ibrutinib 420 mg once daily, 10/16 were receiving 280 mg once daily, and 3/16 were receiving 140 mg once daily. The median duration of treatment with ibrutinib at a reduced dose was not reached (range: 8.4–84.0+ months) for patients who were on ibrutinib treatment for ≥5 years compared with 36.1 months (range: 0.0–84.0+) in all ibrutinib-treated patients with dose reductions (*n* = 31).

Following dose reduction, 13/16 patients (81%) had a resolution of the initial AE. Three patients had AEs that were not resolved at data cutoff (grade 3 malignant lung neoplasm, grade 2 fatigue, and grade 1 contusion in 1 patient each). When considering the subset of AEs for which dose reductions are recommended in the updated ibrutinib US prescribing information (as of May 2022), such AEs led to dose reductions in 4/79 patients (5%) (Table 3). Among these patients, AEs did not recur or recurred at a lower grade in 3/4 patients; 1 patient had recurrence at the same grade AE 3 years after initial resolution (grade 3 atrial fibrillation), that resolved without further dose reduction.

Patients who were on ibrutinib treatment for <5 years (*n* = 56) experienced similar rates of AEs leading to dose reduction (15/56; 27%). Most common reasons for dose reduction by system organ class in this subgroup were hematologic (*n* = 3), cardiac (*n* = 3), and dermatologic (*n* = 3). Dose reductions were more common in response to grade 3 AEs (*n* = 8); however, 100% of AEs (15/15) were initially resolved. Six patients (40%) experienced a recurrence of their AE at the same or higher grade.

AEs led to dose holds of ≥7 days in 45/79 patients (57%) who were on ibrutinib treatment for ≥5 years and in 79/135 patients (59%) in the overall population of all ibrutinib-treated patients (Table 4).

Among patients who were on ibrutinib treatment for ≥5 years, ibrutinib was restarted at 420 mg once daily after dose holds of ≥7 days for most patients (42/45 patients). Following a dose hold of ≥7 days, 43/45 patients (96%) had resolution of the initial AE.

Among patients who were on ibrutinib for ≥5 years, the frequency of AEs leading to dose reductions was highest in years 0–2 and lower in subsequent years, whereas the frequency of AEs leading to dose holds of ≥7 days remained relatively consistent across the first 6 years of treatment (Supplementary Figure S2).

### 3.6. Exploratory Post Hoc Analysis of Outcomes in Patients with Dose Reductions

**Figure 4:**
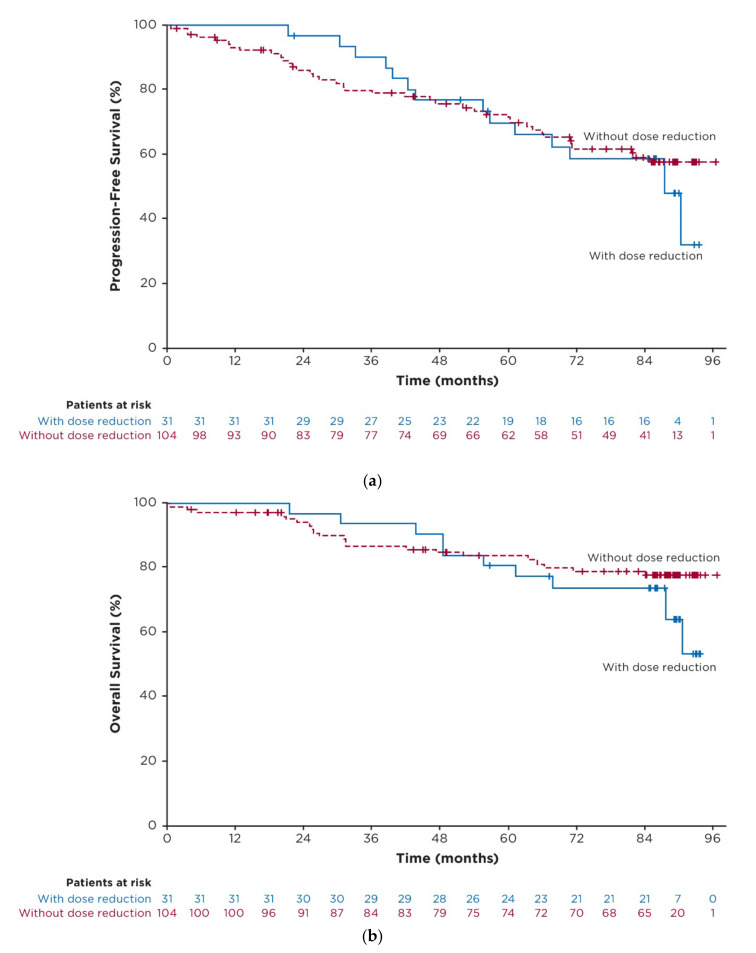
Efficacy in patients with and without dose reductions because of AEs in the overall population of ibrutinib-treated patients. (**a**) Kaplan-Meier curve of investigator-assessed PFS; (**b**) Kaplan-Meier curve of OS. Abbreviations: AE, adverse event; OS, overall survival; PFS, progression-free survival.

In the overall population of all ibrutinib-treated patients, median PFS for patients who had dose reductions (*n* = 31) was 87.7 months (95% CI: 56.9–NE) and was not reached (95% CI: 81.9–NE) for those without dose reductions (*n* = 104) (hazard ratio 0.96 [95% CI: 0.50–1.84]; *p* = 0.9011; Figure 4a). Estimated 7-year PFS rates were 59% (95% CI: 39–74) and 59% (95% CI: 48–68) for patients with and without dose reductions, respectively. With up to 8 years of follow-up, median OS was not reached in either group (hazard ratio 1.28 [95% CI: 0.58–2.83]; *p* = 0.5363; Figure 4b); estimated 7-year OS rates were 74% (95% CI: 54–86) and 79% (95% CI: 69–86) in patients with and without dose reductions, respectively.

### 3.7. Concomitant Medications

Concomitant medications of clinical interest in patients who were on ibrutinib treatment for ≥5 years are shown in Supplementary Table S1. Anticoagulants and antiplatelet agents were frequently used during the treatment period (33% and 65%, respectively), as were antihypertensive medications, including agents acting on the renin-angiotensin system (61%), beta-blocking agents (46%), calcium channel blockers (35%), and other antihypertensives (10%). Overall, 67% of patients received medications to treat acid-related disorders, including proton pump inhibitors in 58% of patients.

## 4. Discussion

Results of the current analysis demonstrate that more than half of patients with previously untreated CLL/SLL were able to receive treatment with single-agent ibrutinib for ≥5 years. While real-world studies have suggested an increased risk of discontinuation of targeted therapies in patients with older age, higher comorbidity burden, higher tumor burden, and/or worse performance status at baseline [13,22,23], no individual baseline characteristics were identified as significant predictors for continuation of long-term ibrutinib treatment in the current study.

Among patients who were on ibrutinib treatment for ≥5 years, responses deepened over time. This subgroup of patients had a higher CR rate over the course of the study (46%) relative to the overall population of ibrutinib-randomized patients (34%) [1], suggesting that patients with favorable responses may be more likely to continue on ibrutinib treatment. In line with this, a higher PFS rate at 7 years was seen in patients who remained on long-term ibrutinib treatment for ≥5 years (82%) relative to the overall ibrutinib-randomized population (59%) [1]. These findings are consistent with those of previous studies, suggesting that continuation of ibrutinib treatment is associated with improved efficacy outcomes [6,7,8].

Safety results in patients who were on ibrutinib treatment for ≥5 years were consistent with those seen in the overall population of ibrutinib-treated patients, including incidences of AEs of clinical interest (hypertension, atrial fibrillation, and major hemorrhage) [1]. AEs generally decreased over time with continued ibrutinib treatment, and no new safety signals emerged in patients who received ibrutinib treatment for ≥5 years. Treatment with ibrutinib was well tolerated irrespective of the frequent use of concomitant antithrombotics, antihypertensives, and acid-reducing agents. Since AEs are the most common reason for discontinuation of ibrutinib in the first-line setting [1,23,24,25,26,27,28], optimization of AE management is crucial to enabling patients to remain on long-term therapy. In the subgroup of patients who were on ibrutinib treatment for ≥5 years, active management of AEs with dose reductions or dose holds was associated with AE resolution in the majority (>80%) of patients. Additionally, dose reductions helped to prevent recurrence or worsening of AEs for most patients, facilitating continued benefit from ibrutinib treatment.

In the current study, disease assessments were performed at regularly scheduled intervals based on iwCLL criteria [20,21]. With up to 8 years of follow-up in the RESONATE-2 study, PFS and OS were similar between patients with and without dose reductions in the overall population of ibrutinib-randomized patients. Patients who had dose reductions received reduced doses of ibrutinib for extended periods of time (median of 3 years in all ibrutinib-treated patients with dose reductions). Together, these results suggest that patients experiencing AEs leading to dose reduction continue to benefit from ibrutinib at the reduced dose. While two real-world studies found significantly worse PFS in patients receiving ibrutinib at reduced doses (<420 mg once daily), this finding from RESONATE-2 is consistent with several other studies that have found no significant difference in efficacy outcomes between patients with dose reductions due to AEs compared to patients without dose reductions [10,12,13,14,15,16,29,30].

## 5. Conclusions

Regardless of demographic and disease characteristics at baseline, more than half (58%) of the patients randomly assigned to the ibrutinib arm in the RESONATE-2 study continued to benefit from ibrutinib treatment for ≥5 years. With up to 8 years of follow-up, the subset of patients who received ibrutinib treatment for ≥5 years experienced sustained efficacy benefits as evidenced by improved depth of response over time and high PFS rates. For patients who received ibrutinib treatment for ≥5 years, the safety profile was consistent with previous reports of long-term ibrutinib treatment and no new or unexpected AEs were observed. Dose modification (dose reduction or dose hold) was effective in resolving AEs for most patients, likely facilitating continuation of ibrutinib treatment.

## Figures and Tables

**Table 1:** Baseline characteristics.

Characteristic	On Ibrutinib Treatment for <5 Years *n* = 57	On Ibrutinib Treatment for ≥5 Years *n* = 79	All Ibrutinib- Randomized Patients *n* = 136
Median age, years (range)	74 (65–89)	71 (65–84)	73 (65–89)
Age group, *n* (%)			
65–69 years	11 (19)	29 (37)	40 (29)
70–74 years	21 (37)	29 (37)	50 (37)
75–79 years	11 (19)	13 (16)	24 (18)
≥80 years	14 (25)	8 (10)	22 (16)
Male sex, *n* (%)	27 (47)	49 (62)	60 (44)
ECOG PS, *n* (%)			
0	27 (47)	33 (42)	60 (44)
1	24 (42)	41 (52)	65 (48)
2	6 (11)	5 (6)	11 (8)
Diagnosis, *n* (%)			
CLL	48 (84)	75 (95)	123 (90)
SLL	9 (16)	4 (5)	13 (10)
Rai stage III/IV, *n* (%)	23 (40)	37 (47)	60 (44)
CIRS score >6, *n* (%)	16 (28)	26 (33)	42 (31)
CrCl <60 mL/min, *n* (%)	28 (49)	32 (41)	60 (44)
Bulky disease ≥5 cm, *n* (%)	25 (44)	29 (37)	54 (40)
LDH			
Median, U/L (range)	215 (65–1188)	196 (52–514)	199 (52–1188)
>250 U/L, *n* (%)	21 (37)	18 (23)	39 (29)
Median β-2 microglobulin, mg/L (range)	5 (2–20)	4 (2–20)	4 (2–20)
Median time from initial diagnosis, months (range)	26 (1–162)	35 (1–241)	30 (1–241)
High-risk features, *n* (%)	28 (49)	45 (57)	73 (54)
*TP53* mutated	4 (7)	7 (9)	11 (8)
del(11q)	11 (19)	18 (23)	29 (21)
Unmutated IGHV	22 (39)	36 (46)	58 (43)
Geographic region, *n* (%)			
United States	12 (21)	19 (24)	31 (23)
Europe	32 (56)	39 (49)	71 (52)
Rest of world	13 (23)	21 (27)	34 (25)

CIRS, Cumulative Illness Rating Scale; CLL, chronic lymphocytic leukemia; CrCl, creatinine clearance; ECOG PS, Eastern Cooperative Oncology Group performance status; IGHV, immunoglobulin heavy chain variable region; LDH, lactate dehydrogenase; SLL, small lymphocytic lymphoma.

**Table 2:** AEs leading to dose reductions per protocol.

AEs Leading to Dose Reductions	On Ibrutinib Treatment for ≥5 Years *n* = 79	All Ibrutinib- Treated Patients *n* = 135
Patients with any AE, *n* (%)	16 (20)	31 (23)
Median time from first dose reduction to discontinuation, months (range)	NR (8.4–87.7)	36.1 (0.0–87.7)
Outcome of first AE leading to dose reduction, *n*/*N* (%) ^a^		
Initial AE resolved	13/16 (81)	28/31 (90)
No recurrence or recurred at lower grade	10/16 (63)	19/31 (61)
Recurred at same or higher grade	6/16 (38)	12/31 (39) ^c^
First dose reduced to, *n*/*N* (%) ^a^		
420 mg to 280 mg	13/16 (81)	27/31 (87)
420 mg to 140 mg	3/16 (19)	4/31 (13)
AEs of interest by SOC, *n* (%) ^b^		
Infection	4 (5)	6 (4)
Hematologic	3 (4)	5 (4)
Dermatologic	2 (3)	4 (3)
Gastrointestinal	1 (1)	4 (3)
Cardiac	1 (1)	2 (1)
Injuries	1 (1)	2 (1)
Musculoskeletal	1 (1)	1 (1)
Neoplasms	1 (1)	1 (1)
Other	4 (5)	9 (7)
Grade of AE, *n* (%) ^b^		
Grade 1	6 (8)	11 (8)
Grade 2	6 (8)	10 (7)
Grade 3	5 (6)	13 (10)
Grade 4	1 (1)	2 (1)

^a^ Denominator is patients with dose reductions because of any AE. ^b^ The same patient may be counted in more than one category because of multiple AE events leading to dose reduction. ^c^ Of 12 AEs that recurred at same/higher grade at any point during treatment, 3/13 were infections, 2/13 were hematologic, 2/13 were cardiac, 1/13 was gastrointestinal, and 4/13 were other. Abbreviations: AE, adverse event; NR, not reached; SOC, system organ class.

**Table 3:** AEs leading to dose reductions per USPI recommendations ^a^.

AEs Leading to Dose Reductions	On Ibrutinib Treatment for ≥5 Years *n* = 79	All Ibrutinib- Treated Patients *n* = 135
Patients with any AE, *n* (%)	4 (5)	11 (8)
Median time from first dose reduction to discontinuation, months (range)	36.1 (8.4–56.0+)	32.9 (0.0–56.0+)
First dose reduced to, *n/N* (%) ^b^		
420 mg to 280 mg	3/4 (75)	9/11 (82)
420 mg to 140 mg	1/4 (25)	1/11 (9)
280 mg to 140 mg	0/4 (0)	1/11 (9)
Outcome of first AE leading to dose reduction, *n*/*N* (%) ^b^		
Initial AE resolved	3/4 (75)	10/11 (91)
No recurrence or recurred at lower grade	3/4 (75)	7/11 (64)
Recurred at same or higher grade	1/4 (25)	4/11 (36) ^c^

^a^ AEs for which dose reductions are recommended in the ibrutinib USPI (grade 2 cardiac failure, grade 3 cardiac arrhythmia, grade 3 or 4 nonhematologic AEs [excluding cardiac failure and cardiac arrhythmia], grade 3 or 4 neutropenia with infection or fever, and grade 4 hematologic AEs). ^b^ Denominator is patients with dose reductions because of AEs per recommendations in the ibrutinib USPI. ^c^ Four patients had AEs that recurred at the same grade (grade 3 atrial fibrillation that recurred after 3 years [*n* = 1], grade 3 pleural effusion that recurred after 2 years [*n* = 1], grade 3 diarrhea that recurred after 4 days [*n* = 1], and grade 3 headache that recurred after 1 week [*n* = 1]). Abbreviations: AE, adverse event; USPI, US prescribing information.

**Table 4:** AEs leading to dose holds ≥7 days.

AEs Leading to Dose Holds ≥7 Days	On Ibrutinib Treatment for ≥5 Years *n* = 79	All Ibrutinib- Treated Patients *n* = 135
Patients with any AE, *n* (%)	45 (57)	79 (59)
Number of dose holds per patient, *n* (%)		
1	20 (25)	35 (26)
≥ 2	25 (32)	44 (33)
Dose restarted after dose hold per patient, *n*/*N* (%) ^a,b^		
420 mg	42/45 (93)	65/79 (82)
280 mg	8/45 (18)	21/79 (27)
140 mg	5/45 (11)	8/79 (10)
Other	0	7/79 (9)
AEs leading to dose hold ≥7 days by SOC, *n* (%) ^a^		
Infection	20 (25)	28 (21)
Neoplasms	8 (10)	13 (10)
Eye disorders	7 (9)	10 (7)
Dermatologic	6 (8)	12 (9)
Gastrointestinal	5 (6)	10 (7)
Injuries	5 (6)	9 (7)
Hematologic	4 (5)	9 (7)
Musculoskeletal	2 (3)	4 (3)
Cardiac	1 (1)	6 (4)
Other	13 (16)	23 (17)
AEs leading to dose hold ≥7 days by grade, *n* (%) ^a^		
Grade 1	6 (8)	10 (7)
Grade 2	25 (32)	38 (28)
Grade 3	30 (38)	49 (36)
Grade 4	5 (6)	12 (9)
AE resolution, *n*/*N* (%) ^b^		
AE resolved with dose hold(s)	43/45 (96)	75/79 (95)
Any AE not resolved	2/45 (4)	12/79 (15)

^a^ The same patient may be counted in more than one category because of multiple AE events leading to dose holds; ^b^ Denominator is patients with dose holds ≥7 days because of any AE. Abbreviations: AE, adverse event; SOC, system organ class.

## Data Availability

Requests for access to individual participant data from clinical studies conducted by Pharmacyclics LLC, an AbbVie Company, can be submitted through Yale Open Data Access (YODA) Project site at http://yoda.yale.edu.

## Supplementary Materials

The following are available online at https://www.mdpi.com/article/10.3390/cancers15020507/s1: Figure S1, Adverse events of clinical interest of any grade by yearly interval; Figure S2, AEs leading to dose modifications over time in patients on long-term ibrutinib treatment for ≥5 years; Table S1, Concomitant medications of clinical interest in patients on long-term ibrutinib treatment for ≥5 years.
