# Supplementary material for: Characteristics and Clinical Outcomes of Patients with Chronic Lymphocytic Leukemia/Small Lymphocytic Lymphoma Receiving Ibrutinib for ≥5 Years in the RESONATE-2 Study
Source: Cancers (Basel). 2023 Jan 13;15(2):507. doi: 10.3390/cancers15020507 (PMC9857192; doi:10.3390/cancers15020507)
Supplement: Supplementary file 1 [file cancers-15-00507-s001.zip › cancers-2102641-supplementary.pdf]

Supplementary Data

Characteristics and Clinical Outcomes of Patients With Chronic Lymphocytic Leukemia/Small Lymphocytic Lymphoma Receiving Ibrutinib For ≥5 Years in the RESONATE-2 Study

Jennifer A. Woyach, Paul M. Barr, Thomas J. Kipps, Jacqueline C. Barrientos, Inhye E. Ahn, Paolo Ghia, Vincent Girardi, Emily Hsu, Mandy Jermain, and Jan A. Burger

Table of Contents

Figure S1. Adverse events of clinical interest of any grade by yearly interval 2

Figure S2. AEs leading to dose modifications over time in patients on long-term ibrutinib treatment for ≥5 years 3

Table S1. Concomitant medications of clinical interest in patients on ibrutinib treatment for ≥5 years 4

Figure S1. Adverse events of clinical interest of any grade by yearly interval.
[a]Combined terms.

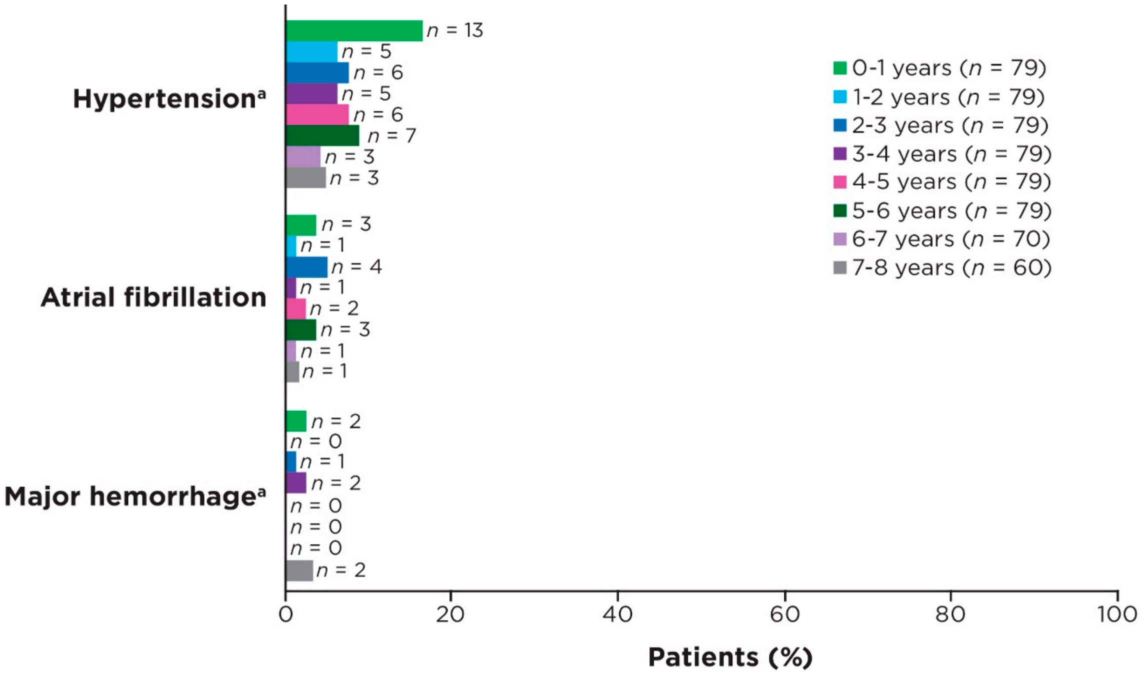

Figure S2. AEs leading to dose modifications over time in patients on long-term ibrutinib treatment for ≥5 years. (**a**) Dose reductions because of AEs of any grade by yearly interval; (**b**) Dose holds ≥7 days because of AEs of any grade by yearly interval. The same patient may appear in more than 1 year. Abbreviation: AE: adverse event.

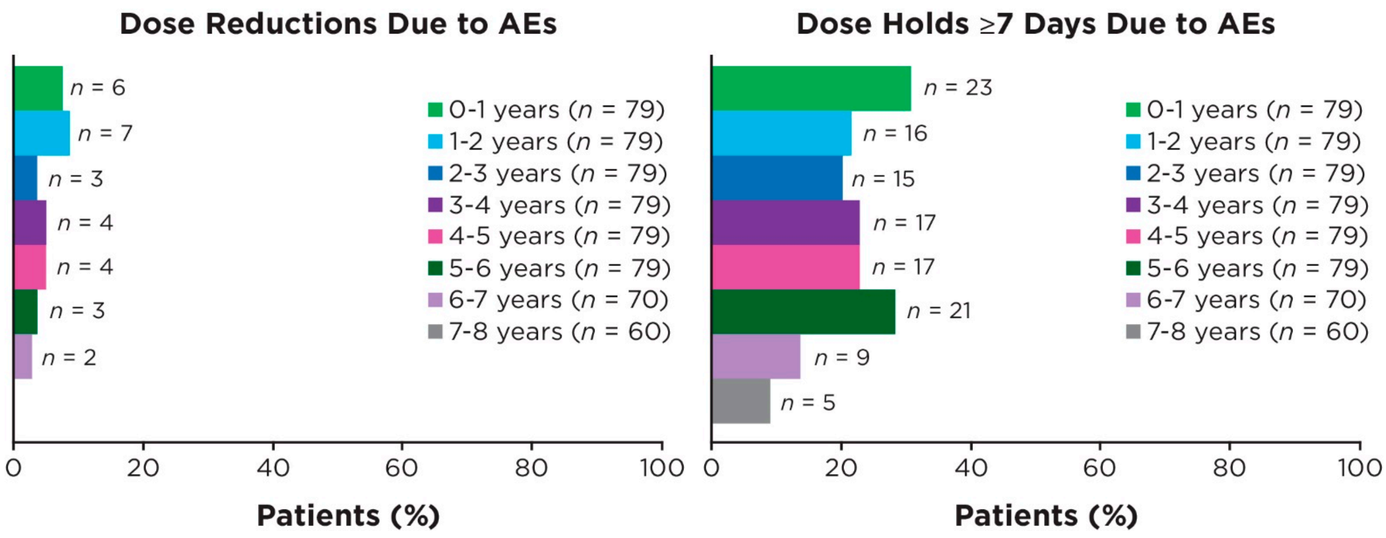

(**a**) (**b**)

Table S1. Concomitant medications of clinical interest in patients on ibrutinib treatment for ≥5 years.

**Concomitant medication, n (%)**	**On ibrutinib treatment for ≥5 years** *n* **= 79**
Antithrombotic agents	59 (75)
Antiplatelets	51 (65)
Anticoagulants	26 (33)
Antihypertensives	
Agents acting on the renin-angiotensin system	48 (61)
Beta-blocking agents	36 (46)
Calcium channel blockers	28 (35)
Other	8 (10)
Drugs for acid-related disorders	53 (67)
Proton pump inhibitors	46 (58)
H2 receptor antagonists	15 (19)
Other	7 (9)
